# Predation on stink bugs (Hemiptera: Pentatomidae) in cotton and soybean agroecosystems

**DOI:** 10.1371/journal.pone.0214325

**Published:** 2019-03-26

**Authors:** Kacie J. Athey, John R. Ruberson, Dawn M. Olson, James D. Harwood

**Affiliations:** 1 Department of Entomology, University of Kentucky, Lexington, Kentucky, United States of America; 2 Department of Entomology, University of Nebraska, Lincoln, Nebraska, United States of America; 3 Crop Protection and Management Research Unit, USDA-ARS, Tifton, Georgia, United States of America; 4 College of Plant Health and Medicine, Qingdao Agricultural University, Qingdao, Shandong, China; University of Catania, ITALY

## Abstract

Stink bugs (Hemiptera: Pentatomidae) are significant pests of cotton and soybeans in the southeastern United States with annual control costs exceeding $14 million in these crops. Three of the most prominent stink bug pests are the southern green (*Nezara viridula*), brown (*Euschistus servus*) and green (*Chinavia hilaris*) stink bugs. To determine trophic linkages between generalist arthropod predators and these pests, species-specific 16S molecular markers were designed and used to detect the presence of prey DNA in predator gut-contents. Over 2700 predators were collected over two growing seasons in cotton and soybean in southern Georgia in 2011 and 2012 and screened for stink bug DNA. Trophic linkages were analyzed relative to prey availability, crop type and field location. The frequency of stink bug DNA in predator guts was negligible on *E*. *servus* (0.23%) and *C*. *hilaris* (0.09%). Overall gut content detection of *N*. *viridula* was 3.3% and *Geocoris sp*. (Hemiptera: Geocoridae), *Orius sp*. (Hemiptera: Anthocoridae) and *Notoxus monodon* (Coleoptera: Anthicidae) were the primary predators. This contrasts with previous studies that reported a much more diverse suite of predators consuming stink bugs with much higher frequency of gut-content positives. The discrepancy between studies highlights the need for replicating studies in space and time, especially if the goal is to implement effective and durable conservation biological control in integrated pest management.

## Introduction

Phytophagous stink bugs (Hemiptera: Pentatomidae) are pests in cotton [1, 2] and soybean crops [3, 4]. Historically, southern green stink bug, *Nezara viridula* (L.), and green stink bug, *Chinavia hilaris* (Say), were the two most important stink bug pests in soybean in the southern United States and South America [3]. However, these two species along with the brown stink bug (*Euschistus servus* (Say)) comprise a stink bug complex in the southern United States [5]. None of these stink bugs are directly affected by Bt (*Bacillus thuringiensis* Berliner) toxins currently used in Bt-transgenic cotton, which is currently in widespread use. Bt-cotton use has led to decreased broad spectrum insecticide use in the southern United States [6]. Further, the cotton boll weevil eradication program also significantly reduced insecticide use [7]. Historically, stink bugs were collaterally controlled by insecticidal sprays targeting other pests, and without these sprays, stink bugs have again emerged as significant pests in southeastern US row-crop systems [8, 9]. Additionally, release from competition with Bt-targeted insects like *Helicoverpa zea* (Boddie) may contribute to stink bug outbreaks in cotton [10].

Traditionally, stink bug species have been lumped together as a pest complex potentially making it difficult to assess their species-specific economic impact [5, 11, 12]. Different species of stink bugs can have differential impacts on cotton, with varied levels of damage to bolls [13], and on soybean crops, in terms of damaged seeds [14], highlighting a need to study stink bugs as individual species and not just as a pest complex. Their wide host ranges and diverse feeding habits complicate the lumping together of the species with respect to their economic impacts. As noted, these stink bug species are pests of soybean and cotton, but are also pests in grain, fruit, nut and vegetable production [15] where they inflict millions of dollars in control costs and yield losses [5], with 2017 losses in Georgia cotton of 47,000 bales, with cost per acre exceeding almost all other pest groups [16]. *Nezara viridula* is highly polyphagous, attacking over 30 species of plants [17–19]. Jones and Sullivan [20] showed that *C*. *hilaris* could utilize about 16 different host plants for development and reproduction. Several other species of stink bugs (e.g. *E*. *servus*, *E*. *tristigmus* (Say), *Thyanta accerra* McAtee) were found to exploit many hosts in addition to economically important crops, such as soybean and cotton [20].

These stink bug species also vary in their susceptibility to insecticides [12, 21]. For example, *E*. *servus* was found to be less susceptible to some pyrethroids and organophosphates than were *C*. *hilaris* and *N*. *viridula* [22]. The variability in species-specific impact on crops, susceptibility to various insecticides, and general ecology underscore the need for an integrated approach to managing stink bug pests.

Integrated pest management (IPM) programs can benefit greatly from incorporating biological control [23]. Generalist predators contribute vital ecosystem services through pest control [24], and pest control utilizing natural enemies in the United States has been estimated to save $4.5 billion annually [25]. Many studies have identified predators of stink bugs with a degree of variability in the results [26–33] (Table 1), but only two recent studies utilized PCR for identifying stink bug predators [34, 35].

**Table 1:** Summary of previous studies on *N*. *viridula*, *C*. *hilaris*, and *E*. *servus* predation.

Predators	Methods	Crop	Reference
Chewing and sucking predators	Sentinel egg masses	Soybean, alfalfa	[33]
Coccinellidae, *Geocoris punctipes*, *Orius insidiosus*, *Podisus maculiventris*, *Solenopsis invicta*, *Nabis roseipennis*, *Lebia analis*	ELISA, eggs	soybean	[28]
Coccinellidae, *Oxyopes salticus*, *Phidippus audax*, *Neoscona arabesca*, *G*. *punctipes*, and *N*. *roseipennis*	ELISA, nymphs	soybean	[28]
Anthicidae, Grasshoppers, phytophagous stink bugs, Chrysopidae larvae, *S*. *invicta*, *Nabis spp*., *Sinea spp*.	Radioactive labeling; visual observations	soybean	[29]
Field predators were not identified	Sentinel egg masses	Weeds, tomato and beans	[26]
Coccinellidae, Formicidae, *Podisus spp*., *Orius spp*., *Geocoris spp*.	Visual observations	Corn, peanut and cotton	[31]
Tettigoniidae, *S*. *invicta*,	Sentinel egg masses	Peanut, cotton and soybean	[27]

Molecular gut-content analysis is a popular tool for determining trophic linkages [36–39] and screening many diverse predators in a short amount of time for a given prey item. This technique is useful in agroecosystems [40, 41] and has been used to help elucidate stink bug food webs [34, 35]. The majority of terrestrial arthropod predators feed cryptically by liquid ingestion following extra-oral digestion [42]. Therefore, determining trophic linkages without molecular methodologies would require visual observations, greatly limiting the number of predators that can be screened quickly. There are however, limitations to molecular gut-content analysis, such as the inability to separate primary predation from secondary predation or scavenging [43]. In addition, molecular gut-content analysis using PCR is a strictly qualitative measure of predation [44]; but if used in conjunction with prey abundance data this method may allow inferences about the potential impact of the predators on the overall pest population [36].

For this project, we employed molecular gut-content analysis to determine which predators from a diverse suite of insects and spiders consumed the three important species of stink bugs in cotton and soybean crops in Georgia. We sampled predators over two years in three locations, and tested for differences in predation as a function of crop types, farm locations and prey availability. The main objective of this study was to determine which predators most frequently preyed upon stink bugs at different times of the season in cotton and soybeans.

## Materials and methods

### Field conditions

Field sampling took place on soybean-cotton farms from July through October 2011 and 2012 in three locations in southwestern Georgia, USA. The locations were USDA-ARS Belflower Farm, Tifton, GA (Tift Co.) (N31° 30.434 W083° 33.430) (planted on 2 June 2011, 17 June 2012), the Attapulgus Research and Education Center, University of Georgia, Attapulgus, GA (Decatur Co) (N30°76.254 W84° 48.488) (planted on 31 May 2011, 17 June 2012) and the Southwest Research and Education Center, University of Georgia, Plains, GA (Sumter Co) (N32° 03.589 W84° 36.691) (planted on 6 June 2011, 17 June 2012). In 2011, three crops at each location were sampled: Bt-cotton (DP1034B2RF), soybeans MG5 (maturity group 5) (Agsouth Genetics 568RR) and soybeans MG6.9 (maturity group 6.9) (Asgrow AG6931RR) (Monsanto Co, St. Louis, MO, USA). The different soybean maturity groups were used because they attract different complexes of predators (McPherson 1996). In 2012, four crops were sampled at each location: Bt-cotton, non-Bt cotton (var. DP147), MG5 soybeans and MG7 soybeans. Aldicarb (Bayer CropScience Leverkusen, Germany), was applied in furrow at planting in cotton (3.93 kg/ha (3.5 lbs/acre)) for thrips control [45]. No other insecticides were applied. Because there were different crop types in the two years, each year was analyzed separately.

### Arthropod sampling

Samples were collected weekly beginning 29 July and ending 30 September in 2011 and 12 July to 11 October in 2012 using a 31 cm diameter sweep net with 100 sweeps per sample, with two samples per field which were pooled for subsequent analyses. We initiated sampling in July each year because stink bugs are not a concern in cotton and soybean until fruit is present on the crops. Within each field, samples were taken along two different rows separated from one another by six rows. Sweeping was initiated five meters into the crop and along rows at least five rows from the plot edge to reduce edge effects. Different rows were sampled on each sample date to prevent prolonged disruption of sampling rows. All arthropods were counted with predators and stink bugs immediately separated and placed in sterile 1.5 mL microcentrifuge tubes filled with 95% ethanol. Specimens were identified to the lowest taxonomic level possible, stink bugs and predatory insects sight identified by J. R. Ruberson and spiders identified by K. J. Athey [46], and then frozen at -20°C until subsequent DNA analysis.

For primer design, specimens of *N*. *viridula*, and *E*. *servus* were collected from laboratory colonies and field locations in Tifton, GA, and non-target species were collected in field locations in Tifton, GA. Primers to amplify *C*. *hilaris* were designed in conjunction with a previous study [47]. Each specimen was preserved as above.

### Molecular gut-content analysis

Total DNA was extracted from all specimens using DNeasy Blood and Tissue Kits (Qiagen Inc., Valencia, CA, USA) following standard animal tissue protocols. For primer design, stink bug legs were removed and DNA was extracted. For molecular gut-content analysis, all predators were crushed and whole body extracted (Table 2).

**Table 2:** List of all predator taxa tested, with numbers testing positive for *N*. *viridula* in PCR testing.

			2011		2012		
Order	Family	Species/Group	PCR (+)	No. Tested	PCR (+)	No. Tested	Reference
Araneae	Anaphyanidae		0	3			
	Araneidae		0	6			[28]
	Linyphiidae		0	10			
	Lycosidae		0	1			[26]
	Lycosidae	*Pardosa sp*.	0	12			
	Oxyopidae		0	12			
	Oxyopidae	*Oxyopes salticus*	1	163	0	174	[26, 28]
	Oxyopidae	*Peucetia virudans*	0	22	0	1	
	Salticidae		0	48			[28]
	Salticidae	*Hentzia sp*.	0	10			
	Salticidae	*Phiddipus sp*.	0	3			
	Salticidae	*Sitticus sp*.	0	14	0	1	
	Tetragnathidae		0	2	0	2	[26]
	Theridiidae	*Latrodectus sp*.	0	1			
	Thomisidae	.	0	48	0	6	
	Thomisidae	*Mesaphesa sp*	0	5			
	Thomisidae	*Misumena sp*.	0	6			
	Thomisidae	*Misumenoides sp*.	0	2			
Blattodea	Blattellidae	*Blattella asahinai*	0	24			^1^[48]
Coleoptera	Anthicidae	*Notoxus monodon*	3	79	13	279	^2^[26, 29]
	Carabidae		0	1			
	Carabidae	*Lebia sp*.	0	31			
	Coccinellidae	*Coccinella septempunctata*			0	5	[26, 31]
	Coccinellidae	*Coleomegilla maculata*	3	9	0	87	[28, 31]
	Coccinellidae	*Harmonia axyridis*	0	15	0	10	[26, 31]
	Coccinellidae	*Hippodamia convergens*	0	66			
Dermaptera	Forficulidae	*Doru taeniataum*	0	1			^1^[49]
	Labiduridae	*Labidura riparia*	0	13			^1^[49]
Hemiptera	Anthocoridae	*Orius spp*.	4	114	39	399	[28, 31]
	Berytidae		0	1			
	Coreidae	*Leptoglossus phyllopus*	0	2			
	Geocoridae	*Geocoris spp*.	5	258	20	564	[26, 28, 31]
	Nabidae	*Nabis sp*.	1	142			[26, 28, 29]
	Pentatomidae	*Podisus maculiventris*	0	21			[28, 31]
	Reduviidae	*Sinea spp*.	0	37			[29]
	Reduviidae	*Zelus spp*.	0	25			[26]
Hymenoptera	Formicidae	*Solenopsis invicta*	0	57			[27]
Orthoptera	Tettigoniidae		0	1			
Neuroptera	Chrysopidae	*Chrysoperla rufilabris*	0	12			[29]; ^3^[26]
	Hemerobiidae	*Micromus sp*.	0	1			

References contain observation evidence justifying inclusion of a given predator taxon

^1^Lepidopteran egg predators

^2^Beetle from the family Anthicidae

^3^Observation during laboratory feeding trials

For primer design, we amplified 16S sequences using general primers; 16Sbr-H and 16Sar-L [50]. Polymerase chain reactions (PCR) (25 μL) consisted of 1X Takara buffer (Takara Bio Inc., Shiga, Japan), 0.2 mM of each dNTP, 0.2 mM of each primer, 1.25 U Takara Ex Taq and template DNA (2 μL of total DNA). PCRs were carried out in Bio-Rad PTC-200 and C1000 thermal cyclers (Bio-Rad Laboratories, Hercules, CA, USA). The PCR protocol was 94°C for 1 min followed by 50 cycles of 94°C for 45 s, 63°C for 45 s, 72°C for 45 s and a final extension of 72°C for 5 min. PCRs included a positive and negative control. Following amplification, the bands were visualized on 2% SeaKem agarose (Lonza, Rockland, Maine, USA) pre-stained with GelRed nucleic acid gel stain (1X; Biotium, Hayward, California, USA). The PCR product was purified and sequenced at AGTC (University of Kentucky, Lexington, KY, USA).

Sequences were edited using Geneious (Biomatters Ltd, Auckland, New Zealand) and aligned using MUSCLE [51]. We designed primers by visually inspecting the sequences using BioEdit 7.0.0 (Isis Pharmaceuticals Inc., Carlsbad, CA, USA) and then using Primer3 [52] to determine whether the primer properties were adequate. PCR reagents were the same as above with PCR protocols of 94°C for 1 min followed by 50 cycles of 94°C for 45 s, 49 or 53°C for 45 s, 72°C for 15 s (Table 3). Following this, the primers were tested against 183 non-targets (S1 Table) for cross reactivity and no amplification was observed. In addition, all primers were target tested against specimens of the respective stink bugs collected from the field with 100% amplification success.

**Table 3:** Primer names and sequences for taxa tested for consumption by predators.

Taxon	Primer Sequence	Amplicon size (bp)	Annealing Temp (°C)	Reference
*N*. *viridula*	NV-334F: 5’-TTTTTATTATTTATTTGGGTTG-3 NV-566R: 5’-GTCGAACAGACCTAGAAC-3’	245	53	Designed herein
*E*. *servus*	ES-43F: 5’-GTCTGATGTTATTTATATCAGATTTAA-3’ ES-295R: -5’-AATAAATATTAACAATTTAACCAAAAC-3’	277	49	Designed herein
*C*. *hilaris*	AH-276F: 5’-AGACCCTATAGAATTTTATTTTAAAG-3’ AH-390R: 5’-CCTAAAAATAATTATATTTAAACC-3’	146	53	[47]

### Statistical analysis

The proportion of predators testing positive for stink bugs was arc-sine square-root transformed to reduce heterogeneity of variance and analyzed by an ANOVA using a generalized linear model in SAS (SAS Institute, Cary, North Carolina, USA). The factors in this analysis were prey availability, week, crop type, and location with interactions between location, crop type and week. For 2011, an ANOVA was run for all predators combined. For 2012, one ANOVA was run for all predators combined and one was run for each of three focal predators. Differences among the locations and crop types were determined by using Tukey’s Honest Significant Difference (HSD) tests. Prey availability was the total number of stink bugs (adults and nymphs combined) of each species collected per field per date (200 sweeps). This number was used to represent the potential prey available to the generalist predators. Total number of nymphs and larvae was used as a proxy for population levels as we did not have information on egg masses, which are the likely prey item for most of our screened predators. Differences between stink bug species capture numbers per year were determined using a repeated measures ANOVA across the season with the average capture number per week of *N*. *viridula*, *C*. *hilaris* and *E*. *servus* as the dependent variable. A Tukey’s HSD test was conducted to determine difference between the means of each stink bug species.

## Results

### Stink bug predation

A total of 2805 predators were tested for predation on stink bugs. Seventeen of 1278 predators tested positive for *N*. *viridula* in 2011 and 72 of 1528 predators were positive for *N*. *viridula* in 2012 (Table 2). 1729 predators were tested for *E*. *servus*, with four individuals testing positive (*Coccinella septempunctata*, *Zelus* sp., *Geocoris* sp., and *Orius* sp.). 2133 predators were tested for *C*. *hilaris*, with 2 individuals testing positive (*Nabis* sp. and *Oxyopes* sp.). Since the frequency of positives for predation on *E*. *servus* and *C*. *hilaris* was negligible, these species were excluded from all other analyses.

### Predation on *N*. *viridula*

**Fig 1:**
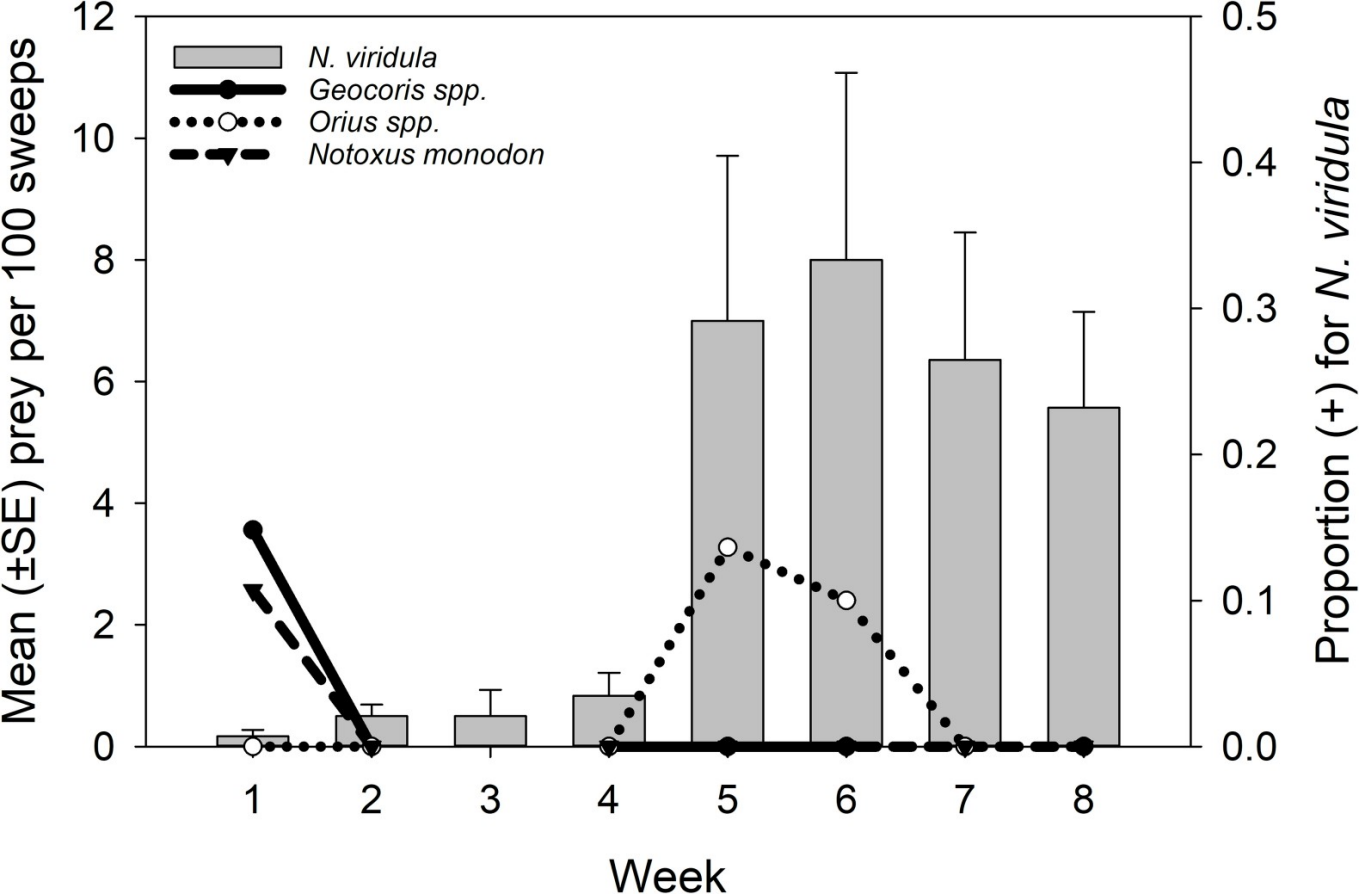
2011 Gut content results. Mean (±SE) number *N*. *viridula* per 100 sweeps and the proportion *Geocoris spp*., *Orius spp*, and *Notoxus monodon* testing positive for *N*. *viridula* DNA by sampling week, 29 July– 7 October 2011. In sampling week 3, only stink bugs were collected.

**Fig 2:**
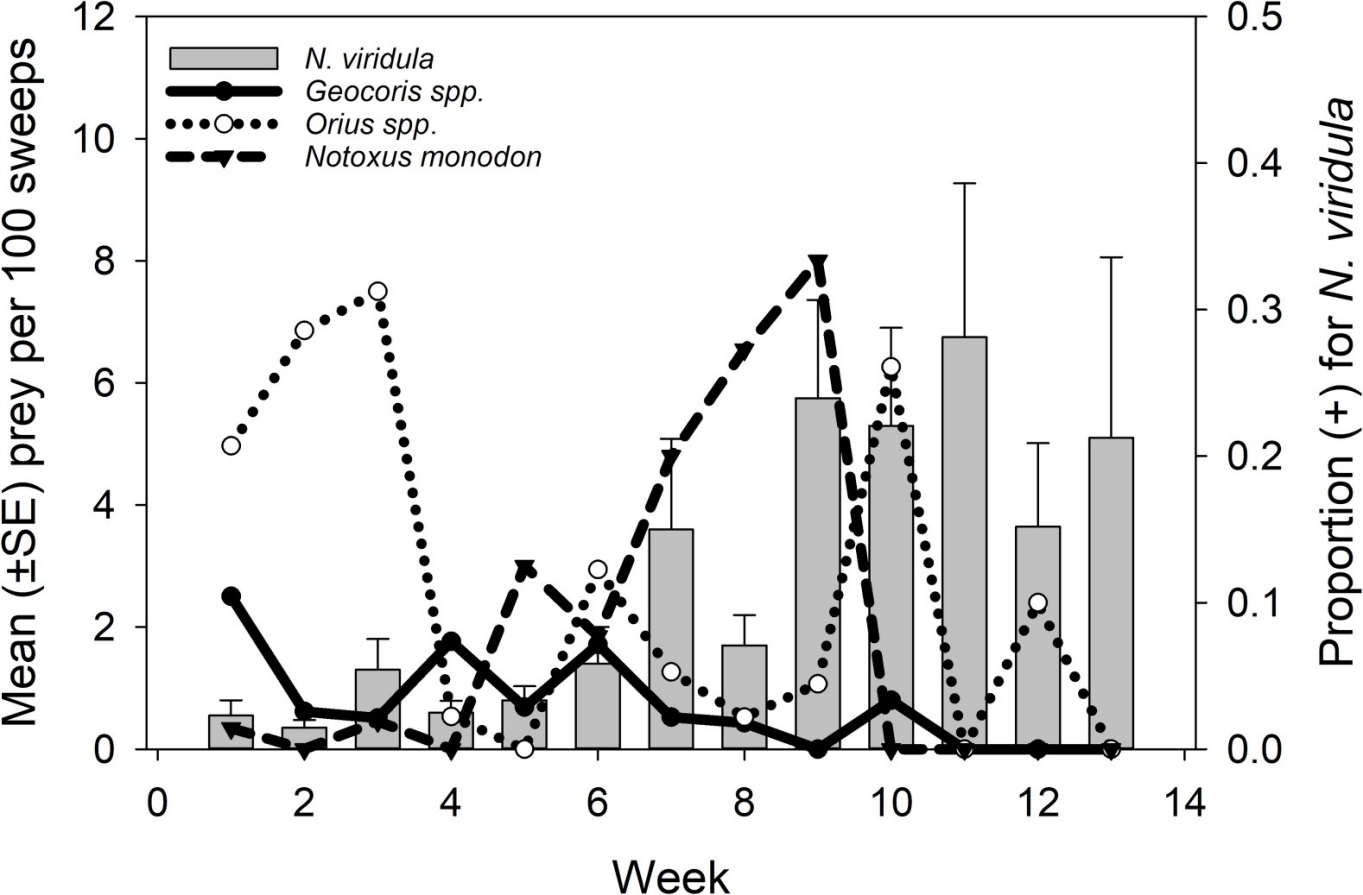
2012 Gut content results. Mean (±SE) number of *N*. *viridula* per 100 sweeps and the proportion *Geocoris spp*, *Orius spp*, and *N*. *monodon*. testing positive for *N*. *viridula* DNA by sampling week, 12 July– 11 October 2012.

The following predators tested positive for *N*. *viridula*: *Coleomegilla maculata* (DeGeer), *Geocoris spp*., *Orius spp*., *Notoxus monodon* (F.), *Nabis spp*., and *Oxyopes spp*. In 2012, only groups that had positives from 2011 were repeated (Table 2). Detected predation in 2011 was very low for all predators with large sample sizes (Fig 1) so frequency of positives by individual predators in 2012 was only analyzed for: *Geocoris spp*. (3.5%, 20/564), *Orius spp*. (9.8%, 39/399), and *Notoxus spp*. (4.7%, 13/279) (Fig 2).

**Fig 3:**
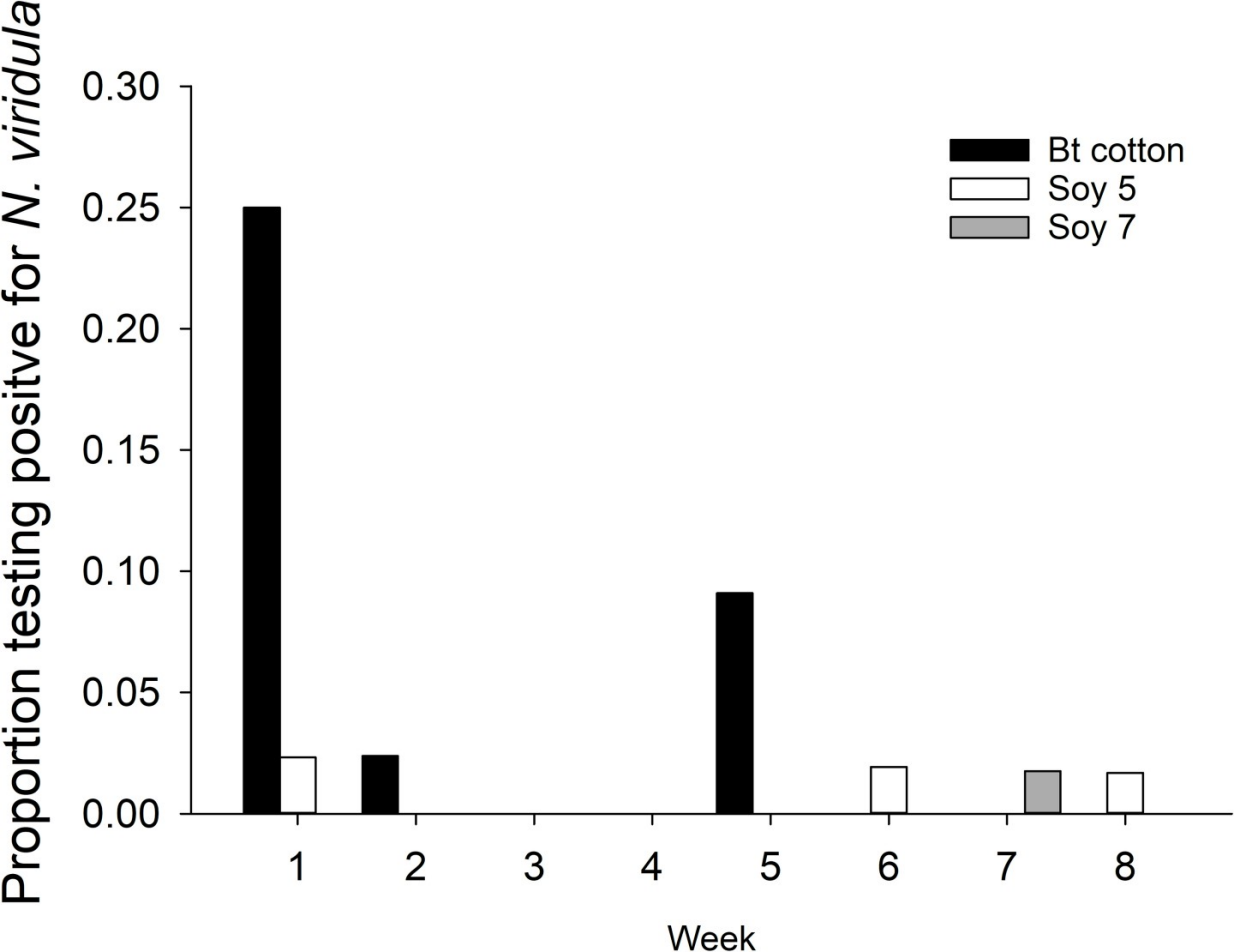
2011 Gut content results by crop. Proportion of combined predators testing positive for *N*. *viridula* DNA by crop and sampling week, 29 July– 7 October 2011. Predators are *Coleomegilla maculata*, *Geocoris spp*., *Orius spp*., *Notoxus monodon*, *Nabis spp*., and *Oxyopes spp*.

When all predators were combined in 2011 (overall model: F_36,12_ = 3.17, p = 0.02) there was a significant interaction of week and crop (F_11, 12_ = 4.00, p = 0.01) (Fig 3). There was higher predation on *N*. *viridula* in Bt-cotton compared to maturity group 7 soybean (Tukey’s HSD, P <0.05) (Fig 3). There was a main effect of location on predation (F_11, 12_ = 3.96, p = 0.05), although no individual differences were detected in the Tukey’s HSD.

In 2012, there were no significant effects of any evaluated variables on frequency of positives (overall model F_78, 29_ = 0.95, p = 0.58). Two of the predator groups, *Nabis spp*. and *Oxyopes spp*., had very low gut-content positives overall and *C*. *maculata* had zero gut-content positives in 2012, so these taxa were not analyzed (Table 2).

In 2012, the overall ANOVA for *Geocoris spp*. predation on *N*. *viridula* was not significant (overall model F_77, 19_ = 1.73, p = 0.09). There was, however, a significant interaction of week and location (F_20, 19_ = 3.17, p = 0.007) on *Geocoris* spp. predation on *N*. *viridula* (Fig 2). Neither the overall ANOVA for *N*. *monodon* predation on *N*. *viridula* (F_18, 40_ = 1.15, p = 0.34) nor the ANOVA for *Orius* spp. predation on *N*. *viridula* was significant (F_18, 22_ = 1.02, p = 0.48).

### Pest population numbers

In 2011, there was no significant difference between the capture numbers of the three stink bug species (F_2, 24_ = 2.30, p = 0.12). In 2012, the overall model was significant (F_2, 39_ = 7.78, p = 0.0014). The number of *N*. *viridula* captured was significantly higher than *C*. *hilaris*. There were however, no significant differences found between either the numbers of *N*. *viridula* and *E*. *servus* captured or *C*. *hilaris* and *E*. *servus* (Tukey’s HSD, P <0.05) (Table 4). Stink bug population numbers never exceeded the economic thresholds in either soybean [53] or cotton [54] when normalized for sampling effort [55].

**Table 4:** Mean stink bugs collected by 100 sweeps by sweep net across the season. Means followed by the same lowercase letter are not significantly different.

Stink Bug Species	2011 Mean ± SE	2012 Mean ± SE
*N*. *viridula*	3.9 ± 1.0 (a)	2.7 ± 0.59 (a)
*C*. *hilaris*	2.17 ± 0.7 (a)	0.1 ± 0.05 (b)
*E*. *servus*	1.6 ± 0.4 (a)	1.2 ± 0.5 (ab)

## Discussion

Of the three stink bug species tested, *N*. *viridula* was the main prey item for generalist predators in our study. *Nezara viridula* also had numerically higher population levels in 2012 compared to the other species. This numerical trend has been observed in other studies in soybean [56] and cotton (personal observation in [8]). Finding several different predators that consume *N*. *viridula* agreed with previous studies on stink bug predation [27–29, 34, 35]. Additionally, the main focal predators we identified, *Geoccoris spp*. and *Orius spp*. have been identified several times by other many other authors (*Geoccoris spp*. [26–28, 34, 35], *Orius spp*. [27, 28, 34, 35].

Predators with diverse feeding modes (sucking and chewing) consuming *N*. *viridula* may be promising for integrated pest management schemes in cotton and soybean. Our study identified several predators with both feeding modes (sucking: *Orius spp*., *Geocoris spp*., and *Nabis spp*.; chewing: *N*. *monodon*, *Oxyopes spp*., and *C*. *maculata*) that were consuming *N*. *viridula*. Although there is considerable observational and molecular evidence for which predators consume stink bugs, the studies vary with predator identity and the impact of these predators in agroecosystems. For predator groups where we had substantial sample sizes, the highest percentage testing positive was 9.8% and this was for *Orius spp*. in 2012. Our study ran for two years and we were never able to detect over ten percent of predators testing positive for any prey group. This low level of positive responses to stink bugs may reflect the availability of alternate prey (including other predators) for the generalist predators surveyed. The general lack of a change in frequency of positive detections in the predators when stink bug populations significantly increased late in the season also suggests that the predators were largely consuming other food resources in each cropping system. In addition, year-to-year variability in predator population density also may have shifted the predator:prey ratio relative to stink bugs and alternate prey. For example, Davis [57] found high variability among years in *Geocoris* spp. density in a peanut agroecosystem, which can affect frequency of predation events.

Our results contrast with two recent field studies employing molecular gut-content analysis to study predation on stink bugs and a related prey item (kudzu bug, *Megacopta cribraria* (F.)) in a cotton-soybean-peanut agroecosystem in the same year and the same region of the US [34, 35]. They found very high percentages of predators testing positive for kudzu bug and stink bugs. They also found many instances of individual predators simultaneously testing positive for kudzu bug and three species of stink bugs. *Geocoris spp*. were especially prone to this in their study, with 4% of *G*. *punctipes* individuals testing positive for four pest species. In contrast, we detected no instances of more than one prey item in a single predator and we had much lower gut-content positives, suggesting possible differences in assay sensitivity. There were also major differences in procedures used. These previous studies were conducted in a single location over a one-month period of a single year [34, 35]. Further, one of their treatments contained buckwheat, a rich source of nectar, which can benefit *Geocoris spp*. [58] although its impact on their population dynamics is unknown. The percent total predators collected that were *Geocoris spp*. in these studies, was higher in soybean, which was adjacent to buckwheat, than in the soybean of our study [34, 35]. In contrast, there were no nectar sources adjacent to our sampling sites and, we sampled at least 5 meters away from the field edges over 8–12 weeks at three well-separated locations, and replicated over two years. Nevertheless, although sampling methods differed, we estimate that the densities of predators and stink bugs in our study were comparable to those in the previous studies [34, 35]. Densities of possible alternative prey in the various cropping systems were not determined in the present or the previous studies, and so we cannot compare possible differences in overall prey availability beyond the stink bug populations. These design and procedural differences likely contributed to at least some of the differences in our findings.

Differences in molecular techniques between studies also could help explain the disparity in the frequency of the same species testing positive for predation. We screened our primers against 183 non-targets from 12 orders and 78 families (Table 3). This contrasts with the non-target testing in other studies with 57 non-targets from 4 orders and 7 families [34] and 83 non-targets from 3 orders and 7 families [35]. Cross amplification of primers can occur across very disparate taxa [59] emphasizing the need for strenuous non-target testing. Additionally, we obtained the primers published in Greenstone et al [34] and tested them against a subset of our non-target extracted DNA (93 non-targets) and found multiple bands in most wells. DNA decay rates also often differ between taxa and primers [60]. Neither our present study, nor the previous two field studies [34, 35] employed decay rate trials and these could help explain the disparity of results. Therefore, differences in primer design among studies may have contributed to differential gut-content amplification.

Variability in predation by omnivores can also occur over years. Omnivorous *Geocoris spp*. have been examined in several studies employing molecular gut-content analysis in open field conditions and estimates of their gut-content positives were quite variable over time. For example, Hagler and Blackmer [40] tested *Geocoris spp*. collected in sweep nets for predation on three different prey items and found that in 2007, 15% of *Geocoris spp*. (N = 215) were found to be preying on *Bemisia tabaci*, whereas in 2008 (N = 160), 46% were positive for *B*. *tabaci*. An opposite trend was observed for *Lygus spp*., with 35% of *Geocoris spp*. testing positive for *Lygus* predation in 2007 and 4% testing positive for *Lygus spp*. in 2008. Therefore, variability in predation over years by omnivorous species may occur because available alternative resources (prey, seed or other food items) as well as due to annual predator population variability [61].

Variability in predation by adults and nymphs of generalist predators within the same crop also can occur. For example, in a study investigating predation of the soybean aphid (*Aphis glycines*) variability was found with *O*. *insidiosus*: 13.4% of adults and 25% of immatures were gut-content positive for *A*. *glycines* [62]. The same trend was found with the other prey species tested—*Neohydatothrips variabilis* (Beach)—with 21.7% of adult *O*. *insidiosus* and 5.0% of immatures positive for *N*. *variabilis* [62]. A study on the same predators and prey, but not separated by life stage, found that *O*. *insidiosus* preyed upon *A*. *glycines* and *N*. *variabilis*, 65% and 35% of the time, respectively [63]. Even within the same system, these generalist predators can vary greatly in their gut-content positives for the same prey items.

The variance in the frequency of a predator species testing positive for the same prey species from year to year, and the potential influence of different densities of predators, available alternative prey, and other food sources on species interactions [40, 62, 63] suggest that the effectiveness of generalist predators and omnivores for controlling stink bug pests may need to be viewed on a case-by-case basis. In addition, our study highlights the need for replicated studies in space and time. Our results contrast with previous studies occurring in the same crops in similar locations [34, 35], but were sampled only one month in a single year. The present study pinpointed several generalist predators that were consuming stink bug pests (*Geocoris spp*., *N*. *monodon* and *Orius spp*.), although they exhibited a low rate of testing positive for these pests. Before implementing a biological control scheme in a specific agroecosystem, it is important to know which natural enemies are having an impact on the focal pests. It is essential, therefore, that experiments exploring the effects of natural enemies on pests be replicated so we can elucidate the variability in and anticipate the outcome of interactions between stink bug populations and their generalist natural enemies. This information will permit optimization of integrated pest management schemes in cotton and soybean agroecosystems addressing stink bugs. A better understanding of the foraging behavior of these predators with a complex cocktail of prey species and densities, and when alternative resources are available, would be needed to be able to predict their biological control potential in relation to a focal pest. And given that the effects of local scale factors on natural enemies can depend on landscape context [64–67], and the wide host range and high dispersal ability of stink bugs, it’s likely important to consider both landscape and local level effects on biological control of these crop pests.

## Supporting information

S1 Table List of non-target taxa screened for cross reactivity with stink bug primers. (DOCX)
