# Supplementary material for: Predation on stink bugs (Hemiptera: Pentatomidae) in cotton and soybean agroecosystems
Source: PLoS One. 2019 Mar 26;14(3):e0214325. doi: 10.1371/journal.pone.0214325 (PMC6435312; doi:10.1371/journal.pone.0214325)
Supplement: S1 Table — (DOCX) [file pone.0214325.s001.docx]

S1 Table. List of non-target taxa screened for cross reactivity with stink bug primers.

| Order | Family | Species | No. Tested |
| --- | --- | --- | --- |
| Araneae | Araneidae | *Magora* sp. | 1 |
|  | Araneidae | *Neoscona crucifera* (Lucas) | 1 |
|  | Araneidae |  | 3 |
|  | Linyphiidae | *Erigone autumnalis* Emerton | 1 |
|  | Linyphiidae | *Glenognatha foxi* (McCook) | 1 |
|  | Linyphiidae | *Tennesseellum formica* (Emerton) | 1 |
|  | Lycosidae |  | 2 |
|  | Miturgidae | *Cheiracanthium* sp. | 1 |
|  | Oxyopidae | *Oxyopes* sp. | 2 |
|  | Salticidae | *Hentzia mitrata* (Hentz) | 1 |
|  | Salticidae | *Pelegrina proterva* (Walckenaer) | 2 |
|  | Salticidae |  | 1 |
|  | Tetragnathidae |  | 2 |
|  | Thomisidae | *Misumena* sp. | 1 |
|  | Thomisidae |  | 1 |
|  | Unidentified |  | 11 |
| Coleoptera | Aderidae |  | 1 |
|  | Anthicidae | *Notoxus* sp. | 2 |
|  | Anthicidae | *Acanthinus argentinus* (Pic) | 1 |
|  | Carabidae | *Lebia viridis* Say | 1 |
|  | Carabidae |  | 2 |
|  | Chrysomelidae | *Diabrotica undecimpunctata* (L.) | 1 |
|  | Chrysomelidae |  | 1 |
|  | Coccinellidae | *Coccinella septempunctata* (L.) | 1 |
|  | Coccinellidae | *Coleomegilla maculata* De Geer | 1 |
|  | Coccinellidae | *Hippodamia convergens* Guérin-Méneville | 1 |
|  | Coccinellidae | *Scymnus* sp. | 1 |
|  | Coccinellidae | *Hippodamia* sp. | 2 |
|  | Coccinellidae | *Coccinella* sp. | 1 |
|  | Curculionidae | *Hypera brunneipennis* (Boh) | 1 |
|  | Curculionidae | *Hypothenemus hampei* Ferrari | 1 |
|  | Elateridae |  | 1 |
|  | Lathridiidae |  | 1 |
|  | Latridiidae |  | 1 |
|  | Meloidae | *Epicauta* sp. | 1 |
|  | Melyridae | *Collops* sp. | 1 |
|  | Nitidulidae |  | 1 |
|  | Phalacridae |  | 1 |
|  | Staphylinidae |  | 2 |
| Diptera | Agromyzidae |  | 1 |
|  | Anthomiidae |  | 1 |
|  | Anthomyzidae |  | 1 |
|  | Brachycera |  | 6 |
|  | Chironomidae |  | 1 |
|  | Chloropidae |  | 1 |
|  | Dolicopodidae |  | 2 |
|  | Drosophilidae | *Scaptomyza* sp. | 1 |
|  | Drosophilidae |  | 2 |
|  | Empididae |  | 1 |
|  | Ephydridae |  | 1 |
|  | Heliomyzidae |  | 1 |
|  | Lonchopteridae |  | 1 |
|  | Muscidae |  | 1 |
|  | Mycetophilidae |  | 1 |
|  | Nematocera |  | 3 |
|  | Phoridae |  | 1 |
|  | Syrphidae |  | 3 |
|  | Tipulidae |  | 1 |
| Hemiptera | Aleyrodidae | *Bemisia tabaci* (Gennadius) | 1 |
|  | Alydidae |  | 3 |
|  | Anthocoridae | *Orius albidipennis* (Reuter) | 1 |
|  | Anthocoridae | *Orius* sp. | 1 |
|  | Aphididae | *Capitophorus eleagni* (Del Guercio) | 1 |
|  | Aphididae | *Uroleucon gravicorne* (Patch) | 1 |
|  | Aphididae |  | 1 |
|  | Cicadellidae |  | 4 |
|  | Coccidae | *Coccus hesperidum* (L.) | 1 |
|  | Coccidae | *Neolecanium cornuparvum* (Thro) | 1 |
|  | Cydnidae | *Sehirus cinctus* (Palisot) | 3 |
|  | Cydnidae |  | 1 |
|  | Geocoridae | *Geocoris* sp. | 4 |
|  | Geocoridae |  | 1 |
|  | Lygaeidae | *Nysius* sp. | 1 |
|  | Miridae | *Lygus lineolaris* (Palisot de Beauvois) | 1 |
|  | Nabidae | *Nabis capsiformis* Germar | 4 |
|  | Nabidae |  | 2 |
|  | Pentatomidae | *Euschistus servus* (Say) | 3 |
|  | Pentatomidae | *Nezara viridula* (L.) | 4 |
|  | Pentatomidae |  | 2 |
|  | Pseudococcidae | *Pseudococcus maritimus* (Ehrhorn) | 1 |
|  | Psyllidae | *Cacopsylla pyricola* (Förster) | 1 |
|  | Psyllidae |  | 1 |
|  | Reduviidae | *Zelus* sp. | 1 |
|  | Reduviidae |  | 2 |
|  | Rhyparochromidae |  | 1 |
|  | Thyreocoridae |  | 1 |
|  | Unidentified |  | 3 |
| Hymenoptera | Argidae |  | 1 |
|  | Bethylidae | *Prorops nasuta* Waterston | 1 |
|  | Bethylidae |  | 1 |
|  | Braconidae | *Aridelus* sp. | 1 |
|  | Braconidae | *Meteorus* sp. | 1 |
|  | Braconidae | *Bracon* sp. | 1 |
|  | Braconidae |  | 5 |
|  | Ceraphronidae | *Aphanogmus* sp. | 1 |
|  | Chalcididae |  | 1 |
|  | Crabronidae | *Mimesa* sp. | 1 |
|  | Crabronidae |  | 1 |
|  | Eulophidae | *Phymastichus coffea* (LaSalle) | 1 |
|  | Figitidae |  | 2 |
|  | Formicidae | *Tapinoma* sp. | 1 |
|  | Formicidae |  | 1 |
|  | Ichneumonidae |  | 3 |
|  | Platygastridae | *Trimorus* sp. | 1 |
|  | Platygastridae |  | 1 |
|  | Pompilidae |  | 1 |
|  | Pteromalidae |  | 1 |
| Lepidoptera | Unidentified |  | 1 |
| Mantodea | Mantidae |  | 1 |
| Neuroptera | Chrysopidae |  | 3 |
|  | Hemerobiidae |  | 2 |
| Orthoptera | Tettigoniidae |  | 1 |
|  | Tettigoniidae |  | 1 |
| Psocoptera | Unidentified |  | 1 |
| Thysanoptera | Thripidae | *Frankliniella occidentalis* (Pergande) | 1 |
|  | Thripidae | *Thrips tabaci* L. | 1 |
| Stylommatophora | Polygyridae | *Mesodon zaletus* (Binney) | 1 |
|  | Discidae | *Anguispira alternata* (Say) | 1 |
